# Impact of two ketogenic diet types in refractory childhood epilepsy

**DOI:** 10.1038/s41390-023-02554-w

**Published:** 2023-03-11

**Authors:** Ali M. El-Shafie, Wael A. Bahbah, Sameh A. Abd El Naby, Zein A. Omar, Elsayedamr M. Basma, Aya A. A. Hegazy, Heba M. S. El Zefzaf

**Affiliations:** 1 Department of Pediatrics, Faculty of Medicine, Menoufia University, Shebin El-Kom, Egypt; 2 Department of Bioinformatics and Medical Statistics, Medical Research Institute, Alexandria University, Alexandria, Egypt

## Abstract

**Background:**

Ketogenic diet (KD) refers to any diet in which food composition induces a ketogenic state of human metabolism.

**Objective:**

To assess short- and long-term efficacy, safety, and tolerability of KD [classic KD and modified Atkins diet (MAD)] in childhood drug-resistant epilepsy (DRE) and to investigate the effect of KD on electroencephalographic (EEG) features of children with DRE.

**Methods:**

Forty patients diagnosed with DRE according to International League Against Epilepsy were included and randomly assigned into classic KD or MAD groups. KD was initiated after clinical, lipid profile and EEG documentation, and regular follow-up was done for 24 months.

**Results:**

Out of 40 patients with DRE, 30 completed this study. Both classic KD and MAD were effective in seizure control as 60% in classic KD group and 53.33% in MAD group became seizure free, and the remaining showed ≥50% seizure reduction. Lipid profile remained within acceptable levels throughout the study period in both groups. Adverse effects were mild and managed medically with an improvement of growth parameters and EEG during the study period.

**Conclusions:**

KD is an effective and safe non-pharmacologic, non-surgical therapy for the management of DRE with a positive impact on growth and EEG.

**Impact:**

- Both common types of KD (classic KD and MAD) are effective for DRE, but unfortunately, nonadherence and dropout rates are frequent.
- High serum lipid profile (cardiovascular AE) is often suspected in children following a high-fat diet, but lipid profile remained in the acceptable level up to 24 months. Therefore, KD constitutes a safe treatment.
- KD had a positive impact on growth, despite inconsistent results of the KD’s effect on growth.
- In addition to showing strong clinical effectiveness, KD also considerably decreased the frequency of interictal epileptiform discharges and enhanced the EEG background rhythm.

## Introduction

Up to 65 million people worldwide are affected by epilepsy.^1^ Approximately one-third of epileptic patients still have difficulty being treated, even though two-thirds of them can control their seizures with current anti-seizure medication (ASM). The International League Against Epilepsy (ILAE) defines drug-resistant epilepsy (DRE) as the failure of adequate trials of two tolerated, properly selected and utilized antiepileptic drug schedules to achieve sustained relief of seizures.^2–4^

The three main objectives of epilepsy treatment are total seizure control, maintaining quality of life (QoL), and avoiding adverse effects (AEs).^2^ The retention rates of ASM can be as low as 50% due to their severe AE, such as a considerable deterioration in QoL.^5^ Therefore, one should initially attempt surgical therapies if a patient is eligible and if surgery is not a possibility for a patient with DRE, vagus nerve stimulation or dietary therapy like the ketogenic diet (KD) are worthwhile alternatives.^6–8^

KD (a high-fat, adequate-protein, and low-carbohydrate diet) is an effective, non-invasive, and non-pharmacologic treatment for refractory childhood epilepsy used since 1920s with few to no neurotoxic effects when compared to multiple ASM.^7,9^ Ketosis (which mimics a starving state), decreased glucose, higher fatty acid levels (which improve bioenergetic reserves), anti-epileptogenic properties and neuroprotective properties are only a few of the various, as-yet-unknown processes through which KD operates.^10^ Also, ketones may confer neurologic protection and ketone bodies could exert anti-oxidative, anti-inflammatory, cellular, epigenetic, and gut-microbiome alterations.^11–13^

Despite the KD’s effectiveness, most patients discontinue the diet because of its unpalatable and restrictive features. So, new variants of KD have emerged, including the modified Atkins diet (MAD) and the low-glycemic-index diet.^14–18^

MAD provides a more palatable and less restrictive dietary treatment option.^14^ The most notable aspect of the MAD is that it begins in an outpatient setting without fasting. The effectiveness of MAD in comparison to classic KD is still debatable. Some studies focused on the major AEs of classic KD in refractory epilepsy, while others showed that MAD is equally effective as classic KD.^15,19,20^

However, since KD is not a physiological diet, it is important to identify and carefully monitor any AEs.^21^ AEs can happen at the start of the diet as well as months after KD initiation. Dehydration, altered electrolytes, hypoglycemia, tiredness, abdominal pain, nausea/vomiting, diarrhea, and constipation are short-term AE, while hypoproteinemia, hypocalcemia, hypercalciuria, urolithiasis, alterations in lipid profiles and increase in transaminases or cardiomyopathy are possible long-term AE.^22,23^ There are few studies available regarding potential long-term negative effects in children using KD for longer than 2 years; the most mentioned symptoms are an increased risk of bone fractures, kidney stones and growth delay.^24^

Electroencephalographic (EEG) features are improved by KD in addition to significantly reducing clinical seizures in epileptic patients. Despite this, there have not been any reported prospective studies of the predictive power of baseline EEG or early changes in EEG for KD therapy response. In addition, few research have compared the electrophysiologic properties of KD responders and non-responders.^25,26^

So, this study aimed to assess short- and long-term efficacy, safety, and tolerability of KD (classic KD and MAD) in childhood DRE and to investigate the effect of KD on EEG features of children with DRE.

## Methods

### Study design

This prospective randomized study was conducted to evaluate the efficacy, safety, and tolerability of the KD and its effect on EEG features among children with DRE.

Our primary outcome was to assess the clinical effectiveness of KD (classic KD and MAD) regarding onset of seizure control, seizure frequency and seizure severity, and to assess long-term safety of KD regarding AE and the effect of KD on growth and lipid profile (cardiovascular risk). The secondary outcome was the evaluation of the effect of KD on EEG features prior to and 3 and 6 months after the KD treatment with the possibility of withdrawal of ASM.

### Study population

We initially enrolled forty patients with DRE attending Pediatric KD outpatient clinic at Menoufia University Hospital from January 2020 to April 2022, after obtaining the approval of the Institutional Review Boards of the Menoufia Faculty of Medicine (ID number 191019 PEDI 29) and an informed (written) consent was obtained from each parent or caregiver.

Our inclusion criteria were patients who had received two or more types of regular antiepileptic drugs, but frequent seizures continued. Patients with chronic diseases, congenital metabolic disorders, liver diseases, and systemic diseases were excluded from the study.

Patients were randomly assigned to two groups, Group 1 (classic KD group): 20 patients received classic KD in the form of formula (Ketocal milk from Danone, Nutricia) and food with the ratio of 3–4 g of fat for every 1 g of carbohydrate and protein, and Group 2 (MAD group): 20 patients kept on MAD consisting of a nearly balanced diet (60% fat, 30% protein, and 10% carbohydrates by weight) providing 100 kcal/kg/day, without restrictions on calories, fluids, protein or need for an inpatient fast and admission.

Before starting KD, we collected demographic and clinical data of studied patients including age, sex, age of start of seizures, anthropometric measurements (weight, length/height, weight for length/height, and BMI) according to Egyptian *Z* score growth references for Egyptian children,^27,28^ etiology, type of seizures, duration of uncontrolled seizures, seizure frequency, and seizure severity [scored according to Chalfont Seizure Severity Scale (CSSS)].^29^ Number of antiepileptic drugs used, and baseline EEG were also documented.

### Method of randomization

The allocation sequence was generated using permuted block randomization technique and the block size was variable.^30^ Allocation sequence/code was concealed from the person allocating the participants to the intervention arms using sealed opaque envelopes.^31^ Double-blinded approach was adopted.^32^ In the present study, consecutive sampling technique was adopted.^33^

### Laboratory procedures

Morning blood samples were taken after 8–12 h fasting. Triglycerides (TG), total cholesterol, low-density lipoprotein (LDL-C) and high-density lipoprotein (HDL-C) were measured. Normal values of laboratory data were considered as follows: serum cholesterol (<170 mg/dL is acceptable, 170–199 mg/dL is borderline and >200 mg/dL is high), serum LDL (<110 mg/dL is acceptable, 110–129 mg/dL is borderline and >130 mg/dL is high) and serum HDL (>45 mg/dL is acceptable, 40–45 mg/dL is borderline and <40 mg/dL is low).^34^

### Initiation of KD

For the first month, carbohydrates were restricted to 10 g/day but were permitted to increase by 5 g/day at intervals of at least 1 month if the child was having difficulty with the restriction of carbohydrates to a maximum of 10% carbohydrates per day by weight. A qualified dietician also educated the parents or caregivers about diet preparation at home with close monitoring of blood glucose and urinary ketones. Multivitamins, calcium, and vitamin D were given as supplements and the antiepileptic drugs and doses used were not changed from those administered previously throughout the study period. Patients were requested to attend their regular monthly outpatient visits for 6 months, then every 3 months for 24 months and the form and frequency of seizures and adverse reactions were observed and recorded.

### KD tolerability

After 1 month of KD initiation, two patients of classic KD group and one patient of MAD group were excluded from the study due to noncompliance of their caregivers. At 3-month follow-up visit, six patients were excluded from the study as they did not tolerate the MAD and the ketogenic liquid formula. Also, one patient of MAD group died secondary to infection in COVID-19 epidemic, so only 30 patients completed the study for 2 years, 15 in each group.

### Short-term outcome

The clinical efficacy of KD was evaluated regarding the onset of improvement of seizures and seizures frequency and severity (according to CSSS) prior to and 3 and 6 months after the KD treatment were analyzed. Effectiveness was evaluated as complete seizure free or seizure reduction percentage change. Percentage change was calculated as follows:

$${{{{{{{\rm{Percentage}}}}}}}}\;{{{{{{{\rm{change}}}}}}}}\left( \% \right) = \frac{{{{{{{\rm{Measurement}}}}}}\;\left( {{{{{{\rm{after}}}}}}} \right) - {{{{{\rm{Measurement}}}}}}\;\left( {{{{{{\rm{before}}}}}}} \right)}}{{{{{{{\rm{Measurement}}}}}}\;\left( {{{{{{\rm{before}}}}}}} \right)}}\times100$$

### KD and EEG

The NIHON KOHDEN EEG-1200K was used to record the EEG in a quiet environment. The 21-channel-EEGs were recorded under typical circumstances (rest, hyperventilation, and photostimulation). Throughout the recording period, children were being studied while resting off with their eyes closed. The International 10–20 system was used to record EEG data from 19 scalp electrodes for 20 min using an average reference (MIZAR-sirius 33 Channels, EBNeuro).^35^

At FP1, F3, C3, P3, O1, F7, T3, T5, FP2, F4, C4, P4, O2, F8, T4, T6, Fz, Cz, and Pz, electrodes were placed. The EEG was digitized at 256 Hz with a time constant of 0.1 sec, a high-frequency filter of 70 Hz and a notch filter in each channel. Based on visual assessment, consecutive, 2-s-long epochs free of artifacts were chosen offline. The provided EEG software was used to perform a rapid Fourier transform on these 20 epochs. Mean-power spectra were gathered for every channel and frequency range in each subject. Six bands were utilized to divide the frequency ranges: Delta (0–4 Hz), Theta (4–8 Hz), Alpha (8–12 Hz), Beta-1 (12–18 Hz), Beta-2 (18–24 Hz), and Gamma (24–64) were the six bands used to separate the frequency ranges.^35^

By comparing EEG before and 1, 3 and 6 months after the KD therapy regarding the background rhythm in the occipital region under the awake and silent states and variations of interictal spike-wave index (SI), the effect of KD on EEG features was assessed. SI represented the mean of the spike discharge times during a second. In the calculation procedure, 100 s of the awake and quiet EEG findings (free of artifact fragments) were chosen, and SI = *n*/100 was used to compute the number of spikes (*n*) present in the recording.

### Long-term outcome

Anthropometric measurements, lipid profile, and the development of AE were monitored at 3-month intervals up to 24 months. In addition, we assessed the possibility of antiepileptic drug withdrawal in seizure free patients after the first 6 months.

### Statistical methodology

Data were collected and entered into the computer using Statistical Package for Social Science program for statistical analysis (ver 25). Data were described using median and interquartile range. Comparisons were carried out between two studied independent not-normally distributed subgroups using the Mann–Whitney U test. Comparisons were carried out among related samples by Friedman’s test. Odds ratio (OR) was used to quantify the strength of the association between two events. McNemar’s test was used on paired nominal data with matched pairs of subjects to determine whether the row and column marginal frequencies were equal. Statistical significance was tested at *P* value < 0.05.

Based on El-Rashidy et al.’s^18^ results, adopting a power of 80% to detect a non-inferiority margin (*d*) of 15% in success rate [complete disappearance of seizures (primary outcome)] and level of significance 5% (*α* = 0.05), the minimum required sample size was found to be 15 patients per group (number of groups = 2) and the total sample size = 30 patients.^36,37^

## Results

Out of 40 patients enrolled initially in our study, 30 patients (13 males and 17 females) with median age 3 years (36 months) in classic KD group versus 6 years (72 months) in MAD group tolerated this study for 24 months, 11 patients (36.67%) were underweight, 3 patients (10%) were overweight, and 7 patients (23.33%) were wasted with non-significant difference between the two groups. Patient characteristics are listed in Table 1.

**Table 1:** Demographic data and anthropometry of studied cases.

	All patients (*n* = 30)	Groups		Test of significance (*P* value)
		Classic KD (*n* = 15)	MAD (*n* = 15)	
Age (months)				
Min–Max	4.00–144.00	4.00–96.00	6.00–144.00	*P* = 0.161 NS
Median [IQR]	48.00 [72.00]	36.00 [48.00]	72.00 [85.00]	
Sex				
Male	13 (43.33%)	6 (40.00%)	7 (46.67%)	*P* = 0.713 NS
Female	17 (56.67%)	9 (60.00%)	8 (53.33%)	
Age of start of seizures (months)				
Min–Max	0.00–96.00	0.00–96.00	2.00–96.00	*P* = 0.036*
Median [IQR]	12.00 [44]	5.00 [21.5]	48.00 [55]	
Weight for age				
Underweight	11 (36.67%)	6 (40.00%)	5 (33.33%)	*P* = 0.70394 NS
Normal	16 (53.33%)	7 (46.67%)	9 (60.00%)	*P* = 0.46540 NS
Overweight	3 (10.00%)	2 (13.33%)	1 (06.67%)	*P* = 0.54186 NS
Length/height for age				
Normal	30 (100.00%)	30 (100.00%)	30 (100.00%)	NA
Weight for length or BMI				
Wasted	7 (23.33%)	4 (26.67%)	3 (20.00%)	*P* = 0.66720 NS
Normal	20 (66.67%)	9 (60.00%)	11 (73.33%)	*P* = 0.44130 NS
Overweight	3 (10.00%)	2 (13.33%)	1 (06.67%)	*P* = 0.54186 NS

*n* number of patients, *Min–Max* Minimum–Maximum, *IQR* interquartile range, *NA* non-applicable statistics.

*Statistically significant (*P* < 0.05); NS: not statistically significant (*P* ≥ 0.05).

### Clinical presentation

According to ILAE (2017),^38,39^ epilepsy of unknown etiology was the commonest etiology among the participants [12 patients (40%)] and Myoclonic seizure was the most prevalent type [10 cases (33.33%)] followed by infantile spasm [6 cases (20%)]. The median duration of uncontrolled seizures before KD was 7 months in classic KD group versus 8 months in MAD group with a median seizure severity scale (according to CSSS) and seizure frequency per day of 165 and 10, respectively. Sixteen patients (53.33%) were on three AEDs, ten (33.33%) patients were on four AEDs and four patients (13.33%) were on two AEDs. The clinical characteristics of the cases before KD are illustrated in Table 2.

**Table 2:** Clinical characteristics before KD.

	All patients (*n* = 30)	Groups		Test of significance (*P* value)
		Classic KD (*n* = 15)	MAD (*n* = 15)	
Type of seizures				
Generalized tonic clonic	5 (16.67%)	2 (13.33%)	3 (20.00%)	*P* = *0.62414 NS*
Myoclonic	10 (33.33%)	5 (33.33%)	5 (33.33%)	*P* = *1.000 NS*
Infantile spasm	6 (20.00%)	3 (20.00%)	3 (20.00%)	*NA*
Focal	3 (10.00%)	2 (13.33%)	1 (06.67%)	*P* = *0.54186 NS*
Unclassified seizures	6 (20.00%)	3 (20.00%)	3 (20.00%)	*NA*
Duration of uncontrolled seizures (months)				
Min–Max	2.00–48.00	2.00–48.00	2.00–36.00	*P* = 0.983 NS
Median [IQR]	7.50 [15.00]	7.00 [11.00]	8.00 [21.00]	
Etiology of epilepsy				
Unknown etiology	12 (40.00%)	8 (53.33%)	4 (33.33%)	*P* = *0.136 NS*
Genetic	7 (23.33%)	3 (20.00%)	4 (33.33%)	*P* = *0.667 NS*
Structural				
Post-anoxic	4 (13.33%)	2 (13.33%)	2 (13.33%)	*NA*
Sturge–Weber syndrome	1 (03.33%)	1 (06.67%)	0 (00.00%)	*P* = *0.3077 NS*
Tuberous sclerosis	1 (03.33%)	0 (00.00%)	1 (06.67%)	*P* = *0.3077 NS*
Miller–Dieker syndrome	1 (03.33%)	0 (00.00%)	1 (06.67%)	*P* = *0.3077 NS*
Post-traumatic	3 (10.00%)	0 (00.00%)	3 (20.00%)	*P* = *0.06724* NS
White matter disease causing leukodystrophy	1 (03.33%)	0 (00.00%)	1 (06.67%)	*P* = *0.3077 NS*
Seizures frequency (baseline) (days)				
Min–Max	00.28–30.00	04.00–30.00	00.28–30.00	*P* = 0.611 NS
Median [IQR]	10.00 [9.00]	10.00 [9.00]	10.00 [11.00]	
Seizures severity scale (baseline)				
Min–Max	111.00–177.00	127.00–177.00	111.00–177.00	*P* = 0.144 NS
Median [IQR]	165.00 [46.00]	169.00 [16.00]	157.00 [54.00]	
Number of antiepileptic drugs				
Two	4 (13.33%)	2 (13.33%)	2 (13.33%)	NA
Three	16 (53.33%)	8 (53.33%)	8 (53.33%)	NA
Four	10 (33.33%)	5 (33.33%)	5 (33.33%)	NA

*NS* non significant, *NA* non-applicable.

### Efficacy of KD

**Fig. 1:**
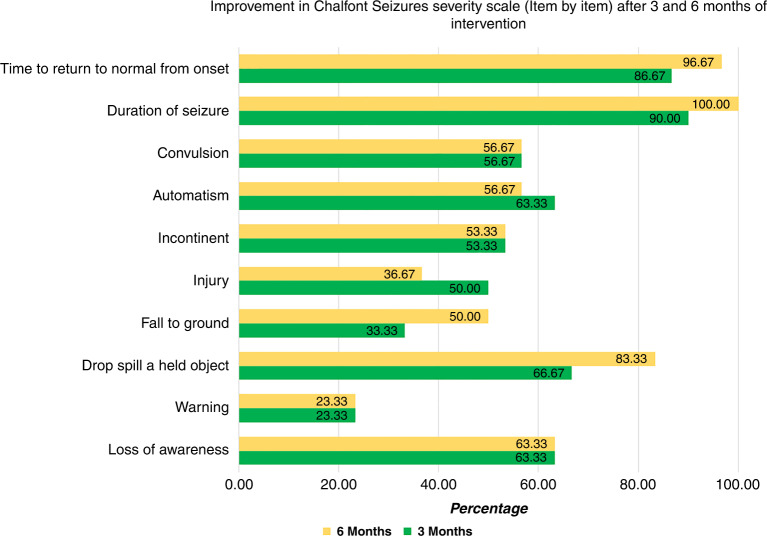
Chalfont seizures severity scale 3 and 6 months after ketogenic diet. Improvement by percentage in Chalfont Seizure Severity Scale (item by item) after 3 and 6 months of intervention.

The onset of seizure improvement was 14 days in classic KD group versus 10 days in MAD group with a non-significant difference between the two groups. Regarding the CSSS, there was a statistically significant decrease in seizure severity by −100% percent change after 3 and 6 months of KD compared to baseline (*P* < 0.0001) without a significant difference between the two groups (Table 3). Analysis of CSSS after 6 months of KD initiation demonstrates improvement of duration of seizures in all patients and improvement in time to return to normal from the onset in 96.67% of patients. Figure 1 illustrates the improvement in CSSS after 3 and 6 months of KD treatment.

**Table 3:** Seizures severity after KD.

	Groups		Test of significance (*P* value)
	Classic KD (*n* = 15)	MAD (*n* = 15)	
Onset of seizures improvement (days)			
Min–Max	07.00–21.00	07.00–21.00	*P* = 0.624 NS
Median	14.00	10.00	
IQR	10.00–14.00	10.00–14.00	
Seizures Severity Scale baseline (at 0 months)			
Min–Max	127.00–177.00	111.00–177.00	*P* = 0.144 NS
Median	169.00	157.00	
IQR	16	54	
Seizures Severity Scale (after 3 months)			
Min–Max	0.00–127.00	0.00–129	*P* = 0.646 NS
Median	0.00	0.00	
IQR	98	111	
Seizures Severity Scale (after 6 months)			
Min–Max	0.00–111.00	0.00–79.00	*P* = 0.963 NS
Median	0.00	0.00	
IQR	70	52	
Friedman test	*χ*^2^_(Fr) (df = 2)_ = 27.882 *P* < 0.0001*	*χ*^2^_(Fr) (df = 2)_ = 27.846 *P* < 0.0001*	
Seizure Severity Scale percentage change (3 M vs baseline)			
*n*	15	15	*P* = 0.680 NS
Min–Max	−100.00 to −24.20	−100.00 to −27.12	
Median	−100.00	−100.00	
IQR	55.37	67.86	
Seizure Severity Scale percentage change (6 M vs baseline)			
*n*	15	15	*P* = 0.909 NS
Min–Max	−100.00 to −34.32	−100.00 to −53.25	
Median	−100.00	−100.00	
IQR	39.55	31.64	
Seizure Severity Scale percentage change (6 vs 3 M)			
*n*	6	7	*P* = 0.086 NS
Min–Max	−74.79 to −3.80	−59.69 to −31.58	
Median	−20.58	−44.88	
IQR	28.90	19.54	

*χ*^*2*^_*(Fr)*_ Friedman Chi-Square, *df* degree of freedom.

Intragroup: statistically significant when compared with baseline values (using Dunn–Sidak method).

*Statistically significant; NS: non significant.

Seizure frequency after 3 and 6 months of KD showed a statistically significant decrease in comparison with baseline (*P* < 0.0001) with a non-significant difference between both groups. Six months after initiation of KD, 60% of patients in classic KD group and 46.67% of patients in MAD group became seizure free and the other 40% and 53.33% had ≥50% seizure reduction with a median percent decrease of −83.33% and −75% after 3 months in classic KD and MAD, respectively, and −100% after 6 months in both groups (Table 4).

**Table 4:** Seizures frequency after KD.

Seizures frequency	Classic KD (*n* = 15)	MAD (*n* = 15)	Test of significance (*P* value)	Total (*n* = 30)
Baseline (days)				
Median [IQR]	10.00 [9.00]	10.00 [11.00]	*P* = 0.611 ΝS	10.00 [9.00]
After 3 months (days)				
Median [IQR]	0.00^♯^ [ΝΑ]	1.00^♯^ [4.86]	*P* = 0.367 ΝS	1.00 [3.00]
After 6 months (days)				
Median [IQR]	0.00^♯^ [ΝΑ]	0.00^♯^ [ΝΑ]	*P* = 0.890 ΝS	0.00^♯^ [ΝΑ]
Friedman test	*χ*^2^_(Fr) (df = 2)_ = 28.000 *P* < 0.0001*	*χ*^2^_(Fr) (df = 2)_ = 25.000 *P* < 0.0001*		
Percentage change (%) (after 3 months)				
Median [IQR]	−83.33 [−50.00]	−75.00 [−43.33]	*P* = 0.831 ΝS	−77.50 [−50.00]
Percentage change (%) (after 6 months)				
Median [IQR]	−100.0 [ΝΑ]	−100.0 [ΝΑ]	*P* = 0.783 ΝS	−100.00 [−33.30]
Seizure frequency reduction after 3 months				
Seizure free (100% reduction)	**8 (53.33%)**	**4 (26.67%)**	***P*** = **0.1362** ΝS	**12 (40.00%)**
SFR (if still convulsions)	**7 (46.67%)**	**11 (73.33%)**		**18 (60.00%)**
50	1 (6.67%)	2 (13.33%)	*P* = 0.54186 ΝS	3 (10.00%)
50–<60	4 (26.67%)	2 (13.33%)	*P* = 0.3628 ΝS	6 (20.00%)
60–<70	1 (6.67%)	3 (20.00%)	*P* = 0.2843 ΝS	4 (13.33%)
70–<80	0 (0.00%)	1 (6.67%)	*P* = 0.3077 ΝS	1 (3.33%)
80–<90	1 (6.67%)	2 (13.33%)	*P* = 0.54186 ΝS	3 (10.00%)
90–<100	0 (0.00%)	1 (6.67%)	*P* = 0.3077 ΝS	1 (3.33%)
Seizure frequency reduction after 6 months				
Seizure free (100% reduction)	**9 (60.00%)**	**7 (46.67%)**	***P*** = **0.46540 NS**	**16 (53.33%)**
SFR (if still convulsions)	**6 (40.00%)**	**8 (53.33%)**		**14 (46.67%)**
50	1 (6.67%)	1 (6.67%)	NA	2 (6.67%)
50–<60	2 (13.33%)	2 (13.33%)	NA	4 (13.33%)
60–<70	1 (6.67%)	2 (13.33%)	*P* = 0.54186 NS	3 (10.00%)
70–<80	1 (6.67%)	2 (13.33%)	*P* = 0.54186 NS	3 (10.00%)
80–<90	1 (6.67%)	1 (6.67%)	NA	2 (6.67%)
90–<100	0 (0.00%)	0 (0.00%)	NA	0 (0.00%)

*SFR* seizure frequency reduction, *NS* non significant, *χ*^2^ (*Fr*) Friedman Chi-Square, *df* degree of freedom.

*Statistically significant.

^#^NA = non applicable.

Bold values refer to the total number of cases who became seizure-free or showed seizure frequency reduction 3 months after KD and followed by details of seizure frequency reduction.

The best seizures control was observed in genetic epilepsy, Sturge–Weber syndrome, tuberous sclerosis, Miller–Dieker syndrome and white matter disease causing leukodystrophy, each of them (11 cases) became seizure free 6 months after KD, followed by cases of post-traumatic epilepsy and post-anoxic epilepsy who responded well to the KD treatment with 2 out of 3 and 3 out of 4, respectively, were seizure free and seizures in the remaining 1/3 and 1/4 of them were reduced by >80–90%. On the other hand, the efficacy of KD for epilepsy of unknown etiology (12 cases) was poor as no patients became seizure free and seizure reduction ranged from >50 to <80%.

In addition to seizure control, we observed that 46.67% of patients in classic KD group and 66.67% of patients in MAD group showed attention improvement 6 months after initiation of KD.

### KD and EEG

**Fig. 2:**
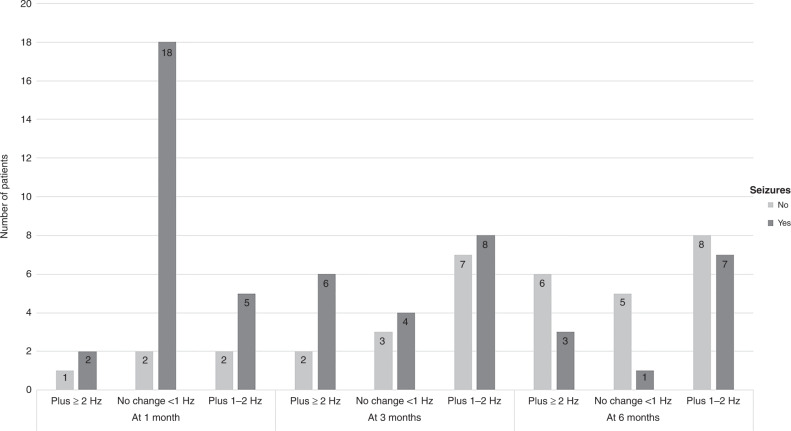
Changes in background rhythm slowing after 1, 3 and 6 months of ketogenic diet.

KD had an impact on the EEG after the first month, and after 3 months, improvements in the background rhythm slowing were observed by plus ≥2 Hz in 8 cases and by plus 1–2 Hz in 15 cases, while after 6 months was observed by plus ≥2 Hz in 9 cases and by plus 1–2 Hz in 15 cases compared to baseline with significant correlation with seizure control after 6 months (*P* value = 0.031 and 0.021), respectively (Table 5). In addition, no change in background rhythm (<1 Hz) which was found in 20 out of 30 cases after 1 month, considerably improved to continue in just 7 cases after 3 months, and in only 6 cases after 6 months, with a significant correlation with seizure control (*P* value < 0.001). Figure 2 illustrates changes in background rhythm slowing after 1, 3 and 6 months of KD.

**Table 5:** EEG changes in response to KD and its correlation with seizure control.

Background rhythm (*n* = 30)	After 1 month		After 3 months			After 6 months		
	No seizures (*n* = 5)	Still seizuring (*n* = 25)	No seizures (*n* = 12)	Still seizuring (*n* = 18)	Test of significance	No seizures (*n* = 16)	Still seizuring (*n* = 14)	Test of significance
Plus ≥2 Hz	1 (33.36%)	2 (66.67%)	2 (25.00%)	6 (75.00%)	*P* = 0.063 NS	3 (33.33%)	6 (66.67%)	***P*** = 0.031*
Plus 1–2 Hz	2 (28.57%)	5 (71.43%)	7 (46.67%)	8 (53.33%)	***P*** = 0.021*	8 (53.33%)	7 (46.67%)	***P*** = 0.021*
No change (<1 Hz)	2 (10.00%)	18 (90.00%)	3 (42.86%)	4 (57.14%)	***P*** < 0.001*	5 (83.33%)	1 (16.67%)	***P*** < 0.001*

Baseline EEG (*n* = 30)	After 1 month			After 3 months			After 6 months		
	No seizures (*n* = 5)	Still seizuring (*n* = 25)	Test of significance OR 95% CI *P* value	No seizures (*n* = 12)	Still seizuring (*n* = 18)	Test of significance OR 95% CI *P* value	No seizures (*n* = 16)	Still seizuring (*n* = 14)	Test of significance
No Epileptiform discharge^(R)^	3 (37.50%)	5 (62.50%)	6.00 0.780–46,145 *P* = 0.085 NS	8 (100.00%)	0 (0.00%)	69.88 3.368–1450.232 ***P*** = 0.006*	8 (100.00%)	0 (0.00%)	29.00 1.480–568.234 ***P*** = 0.026*
Epileptiform discharge	2 (9.09%)	20 (90.91%)		4 (18.18%)	18 (81.82%)		8 (36.36%)	14 (63.64%)	

Spike Index reduction (*n* = 22)	After 1 month		After 3 months			After 6 months		
	No seizures (*n* = 5)	Still seizuring (*n* = 17)	No seizures (*n* = 12)	Still seizuring (*n* = 10)	Test of significance	No seizures (*n* = 16)	Still seizuring (*n* = 6)	Test of significance
>75%	1 (33.33%)	2 (66.67%)	4 (50.00%)	4 (50.00%)	*P* = 0.063 NS	7 (77.78%)	2 (22.22%)	***P*** = 0.031*
>50–75%	1 (20.00%)	4 (80.00%)	4 (66.67%)	2 (33.33%)	*P* = 1.000 NS	5 (71.43%)	2 (28.57%)	*P* = 0.754 NS
30–50%	1 (16.67%)	5 (83.33%)	2 (40.00%)	3 (60.00%)	*P* = 0.687 NS	3 (75.00%)	1 (25.00%)	*P* = 0.687 NS
<30%	2 (25.00%)	6 (75.00%)	2 (66.67%)	1 (33.33%)	*P* = 0.250 NS	1 (50.00%)	1 (50.00%)	***P*** = 0.031*

^*(R)*^ reference category, *CI* confidence interval, *OR* odds ratio, *P* *P* value of McNemar test (compared with 1-month findings), *n* number, *NS* non significant, *Hz* Hertz.

*Statistically significant.

There was a statistically significant difference regarding seizures control between cases with baseline EEG with no epileptiform discharge and cases with baseline EEG with epileptiform discharge after 3 and 6 months of KD as all 8 cases with baseline EEG with no epileptiform discharge became seizures free 3 months after KD compared to cases with baseline EEG with epileptiform discharge only 4/22 and 8/22 became seizures free at 3 and 6 months (*P* value = 0.006 and 0.026) respectively. Moreover, the interictal SI among cases with baseline EEG with epileptiform discharge (22 cases, 11 in each group) showed a significant reduction of >50% after 1 month in 8/22 then this increased to 14 cases after 3 months and 16 cases after 6 months. Also, SI reduction <30% significantly reduced from 8 cases after 1 month to only 2 cases after 6 months with a significant correlation with seizure control (*P* = 0.031). The details of EEG changes in response to KD and its correlation with seizure control are illustrated in Table 5.

### KD and ketosis

Our results did not find any correlation between the level of urinary ketones and seizure control 3 and 6 months after KD with a *P* value of 0.197 and OR 0.300.

### Adverse effects

Our results revealed that gastrointestinal (GI) complications were frequent in our studied cases and constipation was the most common with the same occurrence in the two groups (33.33%), followed by diarrhea with a lower occurrence in classic KD group (13.33%) than MAD group (20%) then vomiting (20%) in classic KD group and (6.67%) in MAD group. We did not document renal/genitourinary complications in our study.

### Long-term outcome

#### KD and cardiovascular risk

**Fig. 3:**
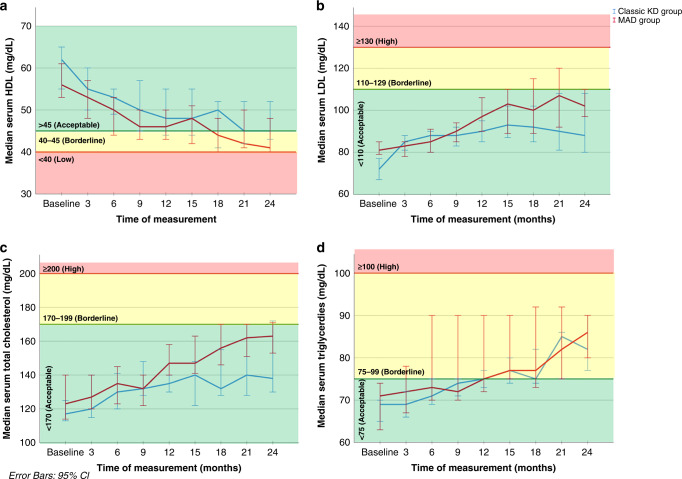
Lipid profile of cases throughout the study (24 months). **a** Simple line graph of median of serum HDL (mg/dL) in the studied groups. **b** Simple line graph of median of serum LDL (mg/dL) in the studied groups. **c** Simple line graph of median of serum total cholesterol (mg/dL) in the studied groups. **d** Simple line graph of median of serum triglycerides (mg/dL) in the studied groups.

In our study, median level of HDL decrease did not reach a low level till the end of the study for 24 months. Also, median level of LDL, total cholesterol, and TG increase did not reach a high level (remained in the acceptable level) throughout the study period, without a significant difference between classic KD group and MAD group. Figure 3 demonstrates serial measurements of lipid profile from baseline up to 24 months.

#### KD and antiepileptic drugs

Upon regular follow-up visits every 3 months after the first 6 months, it was possible to withdraw one AEDs in three patients (20%) of classic KD group and five patients (33.33%) of MAD groups, in addition to withdrawal of two AEDs in three patients (20%) in classic KD group and two patients (13.33%) in MAD group leading to less AEDs side effects and better QoL while on adequate seizure control by KD.

#### KD and growth

**Fig. 4:**
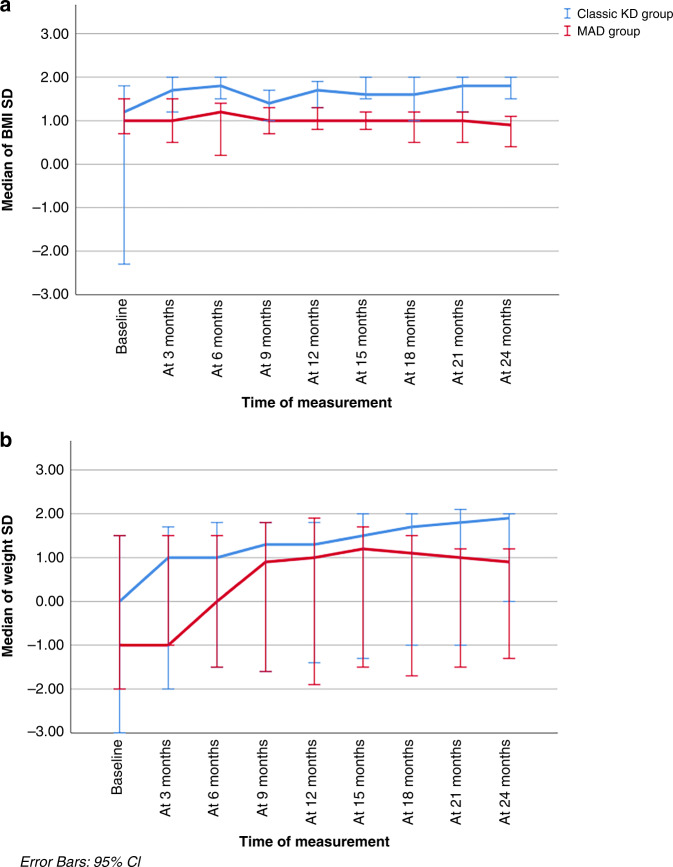
Weight and body mass index of cases throughout the study (24 months). **a** Simple line graph of median (95% CI) of BMI SD in the studied groups. **b** Simple line graph of median (95% CI) of weight SD in the studied groups.

The current study found a positive impact of KD on growth of studied cases (weight loss and BMI reduction were minimal) except in patients who were significantly overweight at diet initiation. Median weight and BMI of cases up to 24 months are illustrated in Fig. 4.

## Discussion

Forty patients with DRE were initially enrolled in this prospective study, but only 30 patients successfully completed it. Six patients (15%) were unable to tolerate KD and were removed from the study (85% tolerability), three additional patients were excluded due to parental noncompliance and one case died during the study period. Overall reasons for dropout were mostly intolerance of the diet, AEs (mostly GI tract related), weight loss, parental unhappiness and change of mind.^40^

Meta-analysis studies have confirmed the efficacy of the KD and showed a seizure frequency reduction (SFR) of ≥50% for both the classic KD and MAD.^41^ Our findings revealed that 60% of patients in classic KD group and 46.67% of patients in MAD group became seizure free and the other 40% and 53.33%, respectively, had ≥50% SFR. Also, the effectiveness of the KD treatment showed an increased tendency over time, with a median decrease in seizure frequency of −83.33% and −100% versus −75% and −100% after 3 and 6 months in the classic KD group and MAD group respectively compared to patients’ baseline.

With a median percent change of −100%, we also documented a statistically significant reduction in seizure severity based on the CSSS 3 and 6 months after the KD compared to baseline in both groups. Two RCTs reported a statistically significant decrease in seizure severity, El-Rashidy et al.^18^ with a mean reduction in seizure severity of 37.63% (MAD) and 35.89% (KD) after 6 months, and Lambrechts et al.^42^ with a mean reduction of 65.2%.

The KD enhanced patients’ cognitive and functional status while also reducing seizure severity and frequency. Six months following KD, we noticed improvements in functional status and cognition, with 66.67% of patients in the MAD group and 46.67% of patients in the classic KD group displaying better attention. This is essential for DRE patients since uncontrolled seizures and the use of numerous anticonvulsants may negatively impact cognition, behavior, drowsiness, memory, and attention issues.^43,44^ These cognitive enhancements might be attributable to the KD given as the mean number of AEDs did not change until 6 months after the diet therapy.

Several studies revealed the effectiveness of KD for epileptic syndromes such as myoclonic-astatic epilepsy, Rett syndrome, West syndrome (particularly combined with tuberous sclerosis), and Dravet and Doose syndromes.^21,45–48^ In our study, all 3 patients with focal seizures, 4/5 of patients with generalized tonic-clonic seizures, 3/6 with infantile spasms, 5/10 with myoclonic seizures and 1/6 with unclassified seizures became seizure free after 6 months after KD initiation with no significant difference between the two groups. However, because of the small sample size, we were unable to draw any conclusions about which type of seizure was linked to better seizure control.

Although the KD has been proven to be a successful treatment for reducing seizures in DRE patients, its wider effects on cerebral neurophysiology are less clear.^49^ KD not only demonstrated good clinical efficacy in this study, but it also significantly reduced the frequency of interictal epileptic discharges and improved the EEG background rhythm. This was evident in 22 patients (73.33%) who had baseline EEG with epileptiform abnormalities (11 in each group) with a reduction in the SI >50% in 8 patients after 1 month, which increased to 14 patients after 3 months and 16 after 6 months. Zhu et al.^50^ reported ≥50% reduction in seizure frequency and reduction of epileptiform discharges in the awake state in 69.0% of patients after 3 months of KD treatment.

KD side effects are frequently blamed for trial dropouts since they are observed in a high percentage of young patients.^7^ More than forty different types of AEs were found with cardiovascular, renal/genitourinary, skeletal systems, and GI (mainly constipation) being the most prevalent.^22,51^ In our study, the most common AEs were GI related, with constipation reported in 33.33% of patients in each group and we did not report any renal/genitourinary AEs in our study. In general, AEs were transient, well controlled by conservative management and did not necessitate for diet discontinuation.

Lipid profile underwent frequent changes throughout the study period. However, HDL median levels did not reach the low level and the median of LDL, total cholesterol and TG measurements remained within the acceptable range till the end of the study for 24 months with a non-significant difference between classic KD and MAD. Kossoff et al., in 2006, 2007, and 2008 over the course of these investigations, observed that there was a comparable increase in total cholesterol and LDL levels, which was within the accepted value.^14,52,53^ Contrarily, Coppola et al.^54^ observed hyperlipidemia as a side effect in their group of patients receiving liquid ketogenic formula for refractory epileptic encephalopathies. Variations of the lipid profile especially during the first 12 months of the diet have been described in up to 60% of children^22^ and may happen during the first month but tend to normalize within the first few months following the diet’s introduction.^24,55–57^ Acceptable change of lipid profile with a long-term duration of KD (24 months) can be a good indicator for the safety of high-fat diet on cardiovascular system in children with DRE.

Systematic reviews have discovered conflicting results about the KD’s effects on growth, with some indicating a favorable benefit and others indicating a negative impact.^55^ Poor caloric and protein intake, acidosis or ketosis, the effects of underlying illnesses and therapies, ambulatory status, and related endocrine changes are some of the etiologies of poor growth in children on KDTs.^58,59^ The current study found that children who were underweight at diet onset [11 (36.67%) underweight and 7 (23.33%) wasted] showed an increase in *Z* scores of child weight over time. However, those who were overweight at diet onset and remained on the diet showed a decrease in the *Z* score over the longer term. This positive impact on growth highlights that fears from KD regarding weight loss were not evident.

Due to their excellent response to the diet, it was possible to withdraw AEDs in 13 patients (43.33%) after the first 6 months: one AEDs in 8 patients (3 in classic KD group vs. 5 in the MAD group) and two AEDs in 5 patients (3 in classic KD group vs. 2 in the MAD group). This can be attributed to the patients’ functional status and QoL.

The study’s points of strength include its prospective nature, which increased the accuracy of our data. We studied two types of KD (classic KD and MAD) commonly used for DRE to allow more options and less diet restrictions. In addition, the effectiveness of the KD regarding seizure control was not measured based solely on caregiver reports but included the EEG changes so allowing more objective evaluation and avoiding subjective errors. Along with growth tracking and documenting of the KD’s effects on growth, we also evaluated the KD’s safety for up to 24 months in terms of side effects and cardiovascular risk.

## Limitation of the study

The main limitations of our study were the small sample size and the heterogeneity of the enrolled patients, which influenced the lack of statistical significance. Another point of limitation was the dropped-out cases due to noncompliance as we started the study with 40 patients and only 30 patients completed the study; the most common reasons for discontinuing the diet were intolerability and poor parental compliance when maintaining the diet.

## Conclusion

With no major side effects reported and a positive influence on EEG and growth, KD (classic KD and MAD) appears to be an effective and generally well-tolerated therapy in the treatment of children with DRE. The role of KD as a single line of therapy, whether from the beginning or after withdrawal of all other medications once full control is established, is also recommended to be clarified by more research.

## Data Availability

All datasets presented in this study are included in the article.
